# Experimental infection of hepatitis E virus induces pancreatic necroptosis in miniature pigs

**DOI:** 10.1038/s41598-020-68959-3

**Published:** 2020-07-21

**Authors:** Soontag Jung, Dong Joo Seo, Daseul Yeo, Zhaoqi Wang, Ae Min, Ziwei Zhao, Mengxiao Song, In-Soo Choi, Jinjong Myoung, Changsun Choi

**Affiliations:** 1 Department of Food and Nutrition, School of Food Science and Technology, College of Biotechnology and Natural Resources, Chung-Ang University, 4726 Seodongdaero, Daedeok-myeon, Anseong-si, Gyeonggi-do 17546 Republic of Korea; 2 Department of Food and Nutrition, Gwangju University, Gwangju, 61743 Republic of Korea; 3 Department of Infectious Disease, College of Veterinary Medicine, Konkuk University, Seoul, 05029 Republic of Korea; 4 Korea Zoonosis Research Institute, Chonbuk National University, Jeonju, 54896 Republic of Korea; 5 Bio and Environmental Technology Research Institute, Chung-Ang University, 4726 Seodongdaero, Daedeok-myeon, Anseong-si, Gyeonggi-do 17546 Republic of Korea

**Keywords:** Virology, Hepatitis

## Abstract

Infection by hepatitis E virus (HEV) via the oral route causes acute hepatitis. Extra-hepatic manifestations of HEV infection may stem from various causes; however, its distribution in organs such as the liver, as well as the mechanisms underlying HEV-induced cell injury, remain unclear. The objective of this study was to determine the chronological distribution of HEV in various tissues of HEV-challenged miniature pigs and to investigate the mechanisms underlying HEV-induced cell death in the pancreas and liver. Virological and serological analyses were performed on blood and faecal samples. Histopathology of the liver and extra-hepatic tissues was analysed. Cell death pathways and immune cell characterisation in inflammatory lesions were analysed using immunohistochemistry. The liver and pancreas displayed inflammation and cellular injury, and a large amount of HEV was observed in the lesions. The liver was infiltrated by T and natural killer cells. HEV was identified in all organs except the heart, and was associated with immune cells. Although the liver and the pancreas strongly expressed TNF-α and TRAIL, TUNEL assay results were negative. RIP3 and pMLKL were expressed in the pancreas. RIP3, but not pMLKL, was expressed in the liver. Pancreatitis induced in HEV-infected miniature pigs is associated with necroptosis.

## Introduction

Worldwide, 14 million symptomatic infections, 5,200 stillbirths, and 300,000 deaths are attributed to hepatitis E virus (HEV) according to the World Health Organisation^1^. While HEV infection is mostly associated with large epidemics in developing countries, autochthonous sporadic cases are on the rise in industrialised countries^2^. In the UK, hepatitis E cases have been increasing since 2009, with an estimated annual infection rate of 200,000, which is largely attributed to the consumption of pork products^3,4^. Clinical symptoms of HEV are mainly associated with acute hepatitis, fulminant hepatic failure, or chronic hepatitis in immunocompromised patients and the elderly. HEV genotype 1 and 2 infection is associated with high mortality rates (up to 25%) in pregnant women^5,6^. Extra-hepatic lesions, including acute pancreatitis, renal failure, neurological diseases, haematological diseases, and the Guillain–Barre syndrome, are linked with HEV infection, cases of which are increasing. However, its pathogenesis is not well understood^7^.

Although several in vivo and in vitro models have been proposed for the purpose of cultivating HEV or mimicking HEV infection in laboratory animals, including rabbits, mice and rats, such in vivo models have not been successful in demonstrating the clinical symptoms of HEV infection^8–10^. The use of non-human primates and conventional pig models is limited by high maintenance costs, difficulties in manipulation, and the need for many personnel^11,12^. Miniature pigs have been experimentally infected with HEV genotypes 1, 3, and 4; they are susceptible to genotypes 3 and 4^13^. Extra-hepatic manifestations have not yet been modelled because existing studies on miniature pig models have focused to the liver. In clinical cases, 55 patients with hepatitis E in India, Nepal, Poland, and France were diagnosed with moderate to severe acute pancreatitis. Although the association between other hepatitis virus infections and acute pancreatitis is well known, the presence of HEV in the human pancreas has not been clearly proven^14,15^. Presently, HEV pathogenesis in the pancreas has not been elucidated in animal models or humans.

HEV infection occurs in humans from all age groups, but mainly in the elderly, who usually suffer from chronic diseases such as hypercholesterolaemia, diabetes, and hypertension. Hepatitis E patients with hypercholesterolaemia are generally prescribed simvastatin, which lowers the blood cholesterol levels and enhances the expression of low-density lipoprotein receptors^16^. As the cholesterol pathway is closely associated with infection by enteric viruses, including HEV and norovirus, treatment with statin drugs may enhance the replication of these viruses^17–19^.

The objective of this study was to analyse the localisation and dynamics of HEV in the liver and other organs in miniature pigs experimentally infected with HEV. HEV-induced cell damage was investigated to unravel the mechanism underlying HEV-induced cell death. In addition, we investigated the effects of simvastatin on HEV infection.

## Results

### Establishment of HEV gt3 infection in miniature pigs

**Figure 1:**
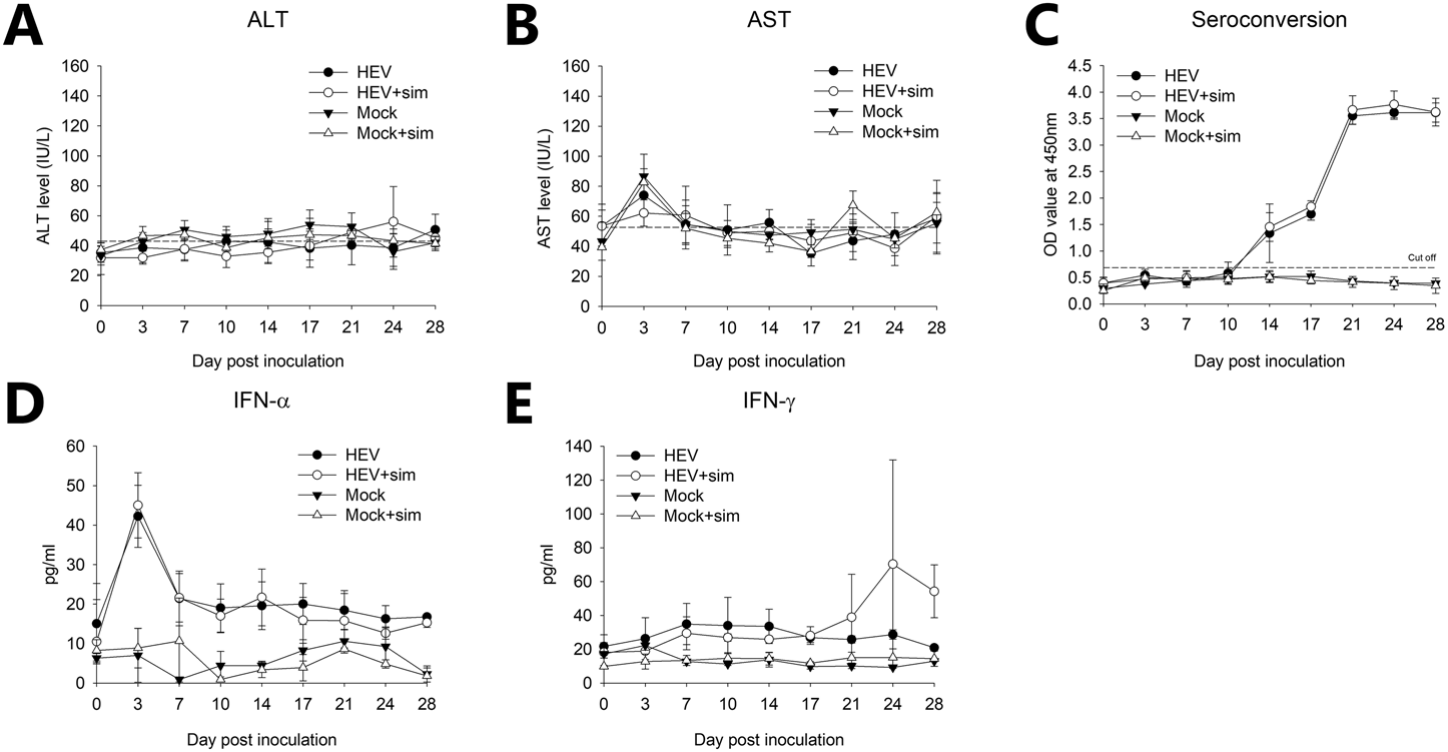
Liver enzyme, seroconversion, and IFN levels in plasma during 28 dpi. (**A**) ALT. Dotted line indicates the normal upper range (ALT 20–48). (**B**) AST. Dotted line indicates the normal upper range (AST 15–53). (**C**) Seroconversion. The cut-off value was calculated as 0.5 plus the mean absorbance of the non-reactive control. (**D**) IFN-α. (**E**) IFN-γ. Results at each time point are expressed as the means ± SDs. *p < 0.05. *HEV* hepatitis E virus, *sim* simvastatin, *IFN* interferon.

HEV RNA was detected in the plasma, peripheral blood mononuclear cells (PBMCs), and faeces by quantitative reverse transcription PCR (RT-qPCR) (Table 1, Supplementary Table S1). HEV RNA was not detected in any of the pre-infection samples, and animals in the two mock groups tested negative throughout the experiment. Faecal virus shedding was the highest at 7 days post infection (dpi) and was observed in 11/12 (91.6%) and 9/12 (75%) of pigs in the HEV group and HEV + sim group, respectively, and gradually decreased, and both groups were not detected at 28 dpi in both groups. Similarly, caecal contents all tested positive until 21 dpi and turned negative at 28 dpi (Supplementary Fig. S1). Although cell-free viraemia was not detected in the two inoculation groups, cell-associated viraemia was detected in 6/12 (50%) and 7/12 (58%) pigs from 3 dpi onwards, and was maintained at more than 50% until 28 dpi. There was no difference in the HEV RNA titre between any of the samples from the pigs in the two groups (Supplementary Fig. S1). Alanine aminotransferase (ALT) and aspartate aminotransferase (AST) levels were not significantly different from those of the mock group (Fig. 1A,B). HEV-specific antibodies were detected in the plasma, indicating that these may be used as an immunological indicator of HEV infection (Fig. 1C). HEV-specific antibodies were first detected at 14 dpi, increased steadily to a maximum at 21 dpi, and remained at this level until the end of the experiment. Interferon (IFN)-α levels were elevated until 3 dpi in both HEV-infected groups, but decreased after 7 dpi and remained low until the end of the experiment (Fig. 1D). The IFN-γ levels increased only in the HEV + sim group, with a significant difference at 28 dpi (Fig. 1E).

**Table 1:** Faecal shedding and cell-free and cell-associated viremia in miniature pigs infected with HEV gt3.

Group	Sample	No. of positive samples/total no. of samples on the indicated dpi							
		3	7	10	14	17	21	24	28
HEV	Plasma	0/12	0/12	0/9	0/9	0/6	0/6	0/3	0/3
	PBMCs	6/12	6/12	7/9	6/9	3/6	3/6	2/3	3/3
	Faeces	4/12	11/12	7/9	8/9	4/6	3/6	1/3	0/3
HEV + sim	Plasma	0/12	0/12	0/9	0/9	0/6	0/6	0/3	0/3
	PBMCs	7/12	8/12	7/9	8/9	4/6	4/6	1/3	3/3
	Faeces	5/12	9/12	6/9	8/9	5/6	4/6	1/3	0/3
Mock	Plasma	0/3	0/3	0/3	0/3	0/3	0/3	0/3	0/3
	PBMCs	0/3	0/3	0/3	0/3	0/3	0/3	0/3	0/3
	Faeces	0/3	0/3	0/3	0/3	0/3	0/3	0/3	0/3
Mock + sim	Plasma	0/3	0/3	0/3	0/3	0/3	0/3	0/3	0/3
	PBMCs	0/3	0/3	0/3	0/3	0/3	0/3	0/3	0/3
	Faeces	0/3	0/3	0/3	0/3	0/3	0/3	0/3	0/3

*HEV* hepatitis E virus, *PBMCs* Peripheral blood mononuclear cells, *dpi* day post inoculation, *sim* simvastatin.

### HEV infection leads to pancreatic injury as well as hepatitis with lymphocytic infiltration

**Figure 2:**
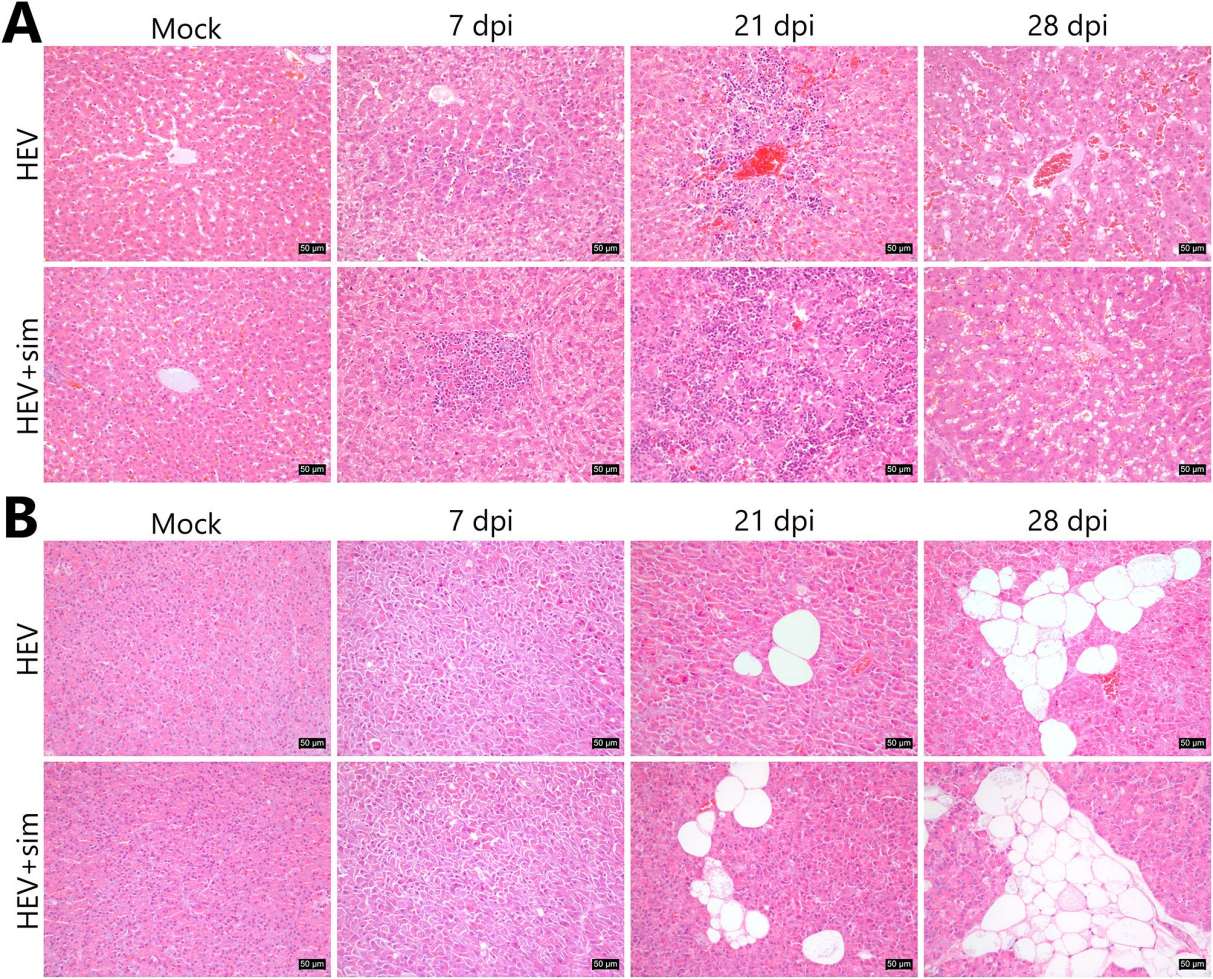
Histopathological alterations in the liver and pancreas. (**A**) Histopathological alterations in liver sections over time, revealing slight to moderate inflammatory cell infiltration and microvesicular steatosis with glycogenated nuclei. (**B**) Histopathological alterations in the pancreatic sections over time, revealing cell membrane rupture, nuclear condensation, nuclear fragmentation, and cell depletion. Tissue sections were stained with haematoxylin and eosin. Scale bars = 50 μm. *HEV* hepatitis E virus, *sim* simvastatin.

Histopathological analysis of liver sections revealed hydropic degeneration and periportal lymphocytic infiltration in some individuals in the HEV group at 7 dpi (Fig. 2A). At 21 dpi, mild multifocal lymphocytic infiltration was observed. At 28 dpi, microvascular steatosis and glycogenated nuclei were present, but lymphocytic infiltration was no longer observed.

**Figure 3:**
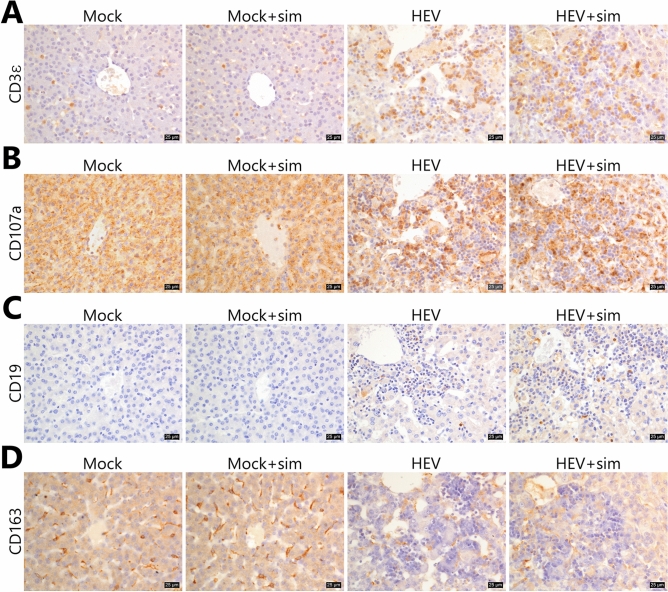
Characterisation of infiltrating immune cells in the liver. (**A**) Representative images of CD3ε staining. (**B**) Representative images of CD107a staining. (**C**) Representative images of CD19 staining. (**D**) Representative images of CD163 staining. Nuclei were counterstained with Meyer’s haematoxylin. Scale bars = 25 μm. *HEV* hepatitis E virus, *Sim* simvastatin.

In HEV group, cell membrane rupture as well as nuclear condensation and fragmentation were observed in a large area of the pancreas at 7 dpi (Fig. 2B). Mild cell vacuolation and cell depletion were observed starting from 21 dpi and peaked at 28 dpi. In the HEV + sim group, similar events as in the HEV group were observed, but they were slightly more severe. Histopathological lesions were not detected in the liver and pancreatic tissues of pigs from the two mock groups. The infiltrating cell profile was dominated by CD3ε as a T-cell marker and CD107a as a marker of functional natural killer (NK) cells, and significantly less so by the B-cell marker, CD19 (Fig. 3A–C). CD163, a macrophage marker that is also expressed in Kupffer cells, was detected in normal areas, but not in infiltrated lesions (Fig. 3D).

**Figure 4:**
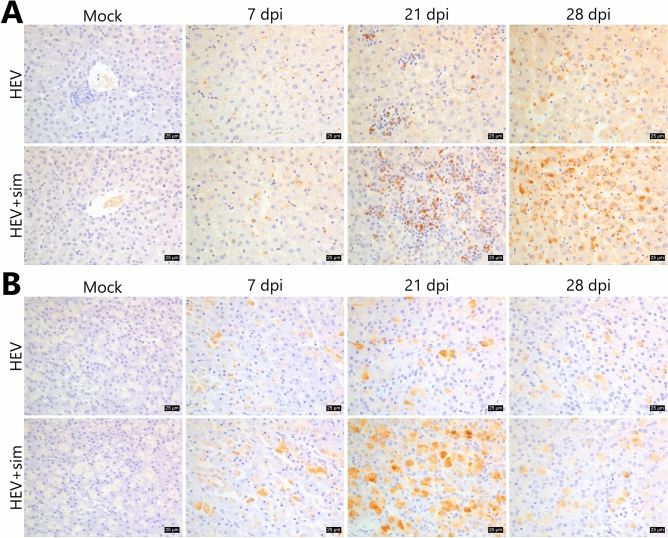
Localisation of HEV in the liver and pancreas. (**A**) Localisation of HEV in the liver sections over time; HEV antigens are distributed in the infiltrated immune cells and the perinuclear regions of hepatocytes. (**B**) Localisation of HEV in the pancreatic sections over time; HEV antigens are dominantly distributed in the acinar cells. Brown colour indicates HEV. Nuclei were counterstained with Meyer’s haematoxylin. Scale bars = 25 μm. *HEV* hepatitis E virus, *sim* simvastatin.

Immunohistochemistry (IHC) identified HEV antigens in the liver (Fig. 4A). HEV antigens were present at low levels mainly in the cytoplasm and perinuclear regions of hepatocytes at 7 dpi. In livers infiltrated by inflammatory cells, HEV antigens were predominantly localised in infiltrated inflammatory cells, rather than in hepatocytes. After the inflammatory cells receded at 28 dpi, HEV antigens were prominently detected in the cytoplasm of hepatocytes. In the pancreas, HEV antigens were exclusively localised in the cytoplasm of acinar cells, and not in the islets of Langerhans (Fig. 4B). On 7 dpi, HEV antigens were detected as spots around the perinuclear regions of damaged cells only, and persisted throughout the cytoplasm of some cells at up to 28 dpi. HEV antigens were not found in or near vacuolated cell lesions. Simvastatin treatment slightly increased HEV antigen in the liver and pancreas, whereas both mock groups displayed no signals.

### HEV enters various organs via infected immune cells

**Figure 5:**
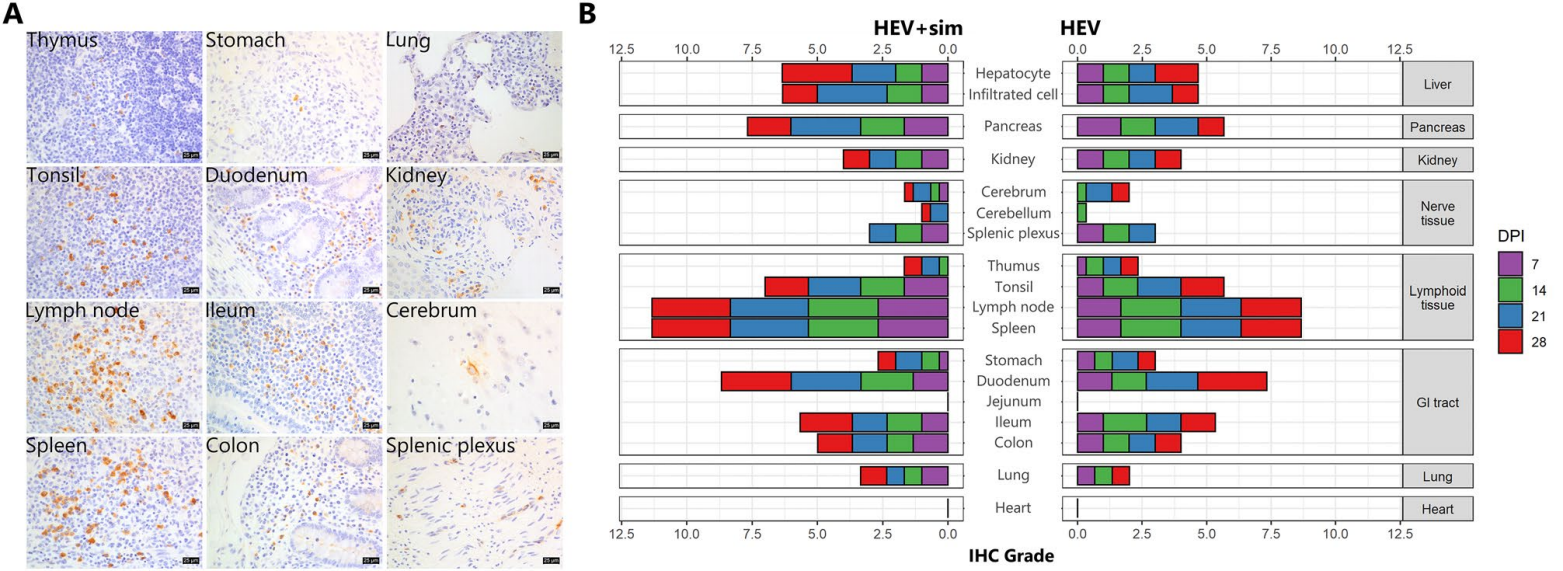
Extra-hepatic localisation of HEV. (**A**) Localisation of HEV in lymphoid tissues, the gastrointestinal tract, lungs, kidneys, and nerve tissues. Brown colour indicates HEV. Nuclei were counterstained with Meyer’s haematoxylin. Scale bars = 25 μm. (**B**) Time course of IHC grade for hepatic and extra-hepatic localization of HEV. HEV-infected cell proportions were scored as follows: − , none; 1, < 5% positive/total area (400 ×); 2, 5–10% positive/total area; 3, > 10% positive/total area. Data represent the mean of each group. *DPI* day post inoculation.

Lymphoid tissues, including those of the tonsils, spleen, and lymph nodes, were continuously monitored from the very early stages of infection until the end of the experiment, and were found to have the highest amounts of HEV antigens. A few cells in the thymus were also positive for HEV (Fig. 5A, Supplementary Fig. S2). In the gastrointestinal tract, including the stomach, duodenum, ileum, and colon, HEV was mainly distributed in the immune cells of the lamina propria or concentrated in lymphoid tissues, such as the Peyer’s patches (Fig. 5A, Supplementary Fig. S3). Interstitial thickenings due to lymphocytic infiltration were observed in the lungs, and HEV antigens were distributed in these lesions and in lymphoid nodules adjacent to the bronchioles (Fig. 5A, Supplementary Fig. S4). Mild inflammatory cell infiltration was observed in the kidneys, where HEV antigens were detected in infiltrated cells in a manner similar to that seen in previously described organs (Fig. 5A, Supplementary Fig. S4). HEV antigens were also present in the glomeruli of some individuals. With regard to the brain and peripheral nerves (splenic plexus), HEV antigens were detected in peripheral nerves at up to 21 dpi and in the neuronal cell bodies and axons in the brain from 21 dpi (Fig. 5A–C, Supplementary Fig. S4, Table S2). HEV was not present in the heart.

### HEV induces necroptosis, which is the major injury mechanism in the pancreas

**Figure 6:**
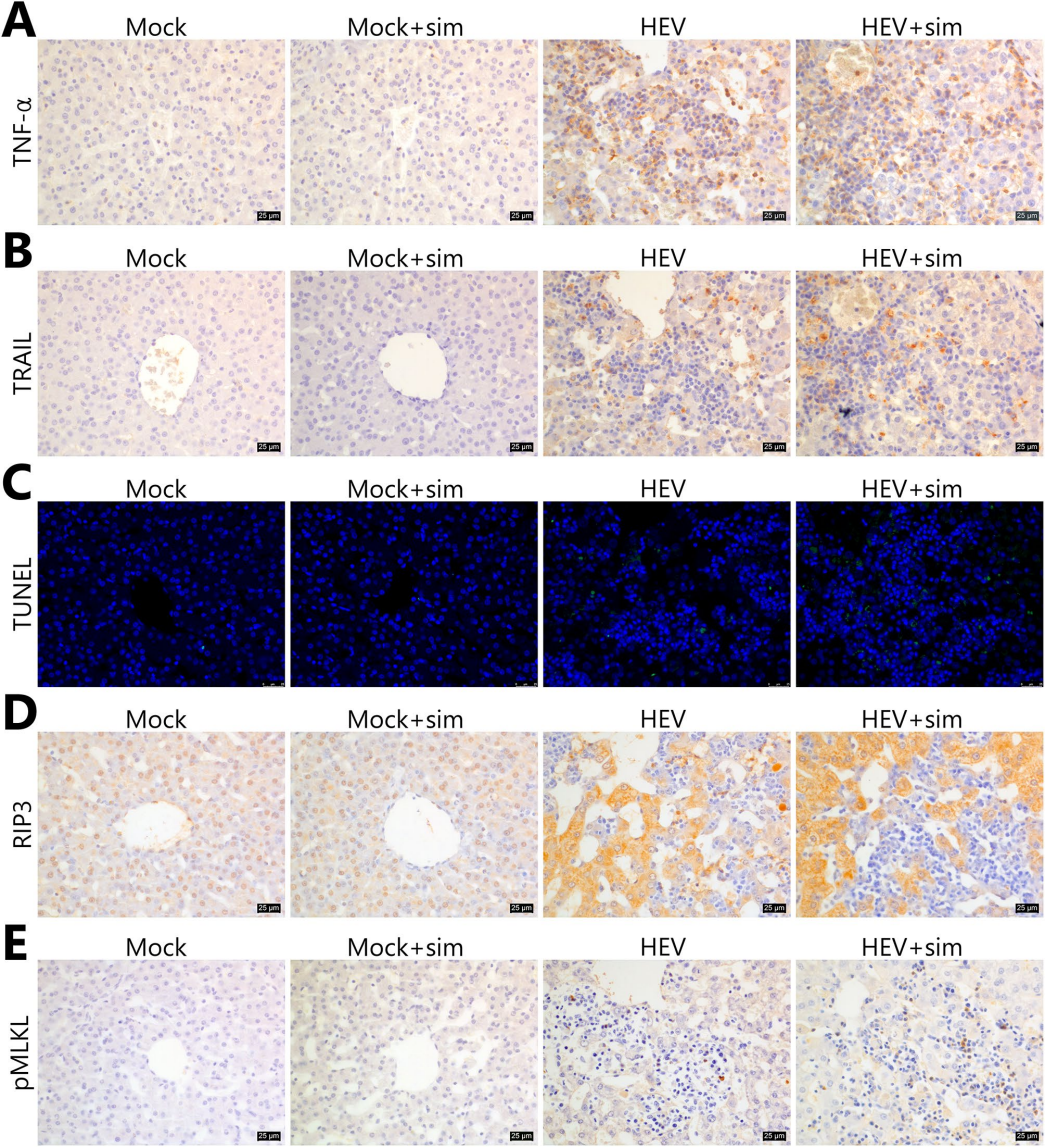
Immunohistochemical analysis of necroptosis in the liver. (**A**) Representative images of TNF-α staining. (**B**) Representative images of TRAIL staining. (**C**) Representative images of TUNEL assay. Green fluorescence indicates DNA fragmentation. Nuclei were counterstained with DAPI. (**D**) Representative images of RIP3 staining. (**E**) Representative images of pMLKL staining. (**A**, **B**, **D**, **E**) Nuclei were counterstained with Meyer’s haematoxylin. Scale bars = 25 μm. *HEV* hepatitis E virus, *Sim* simvastatin, *TRAIL* TNF-related apoptosis-inducing ligand, *TUNEL* terminal deoxynucleotidyl transferase dUTP nick-end labelling.

**Figure 7:**
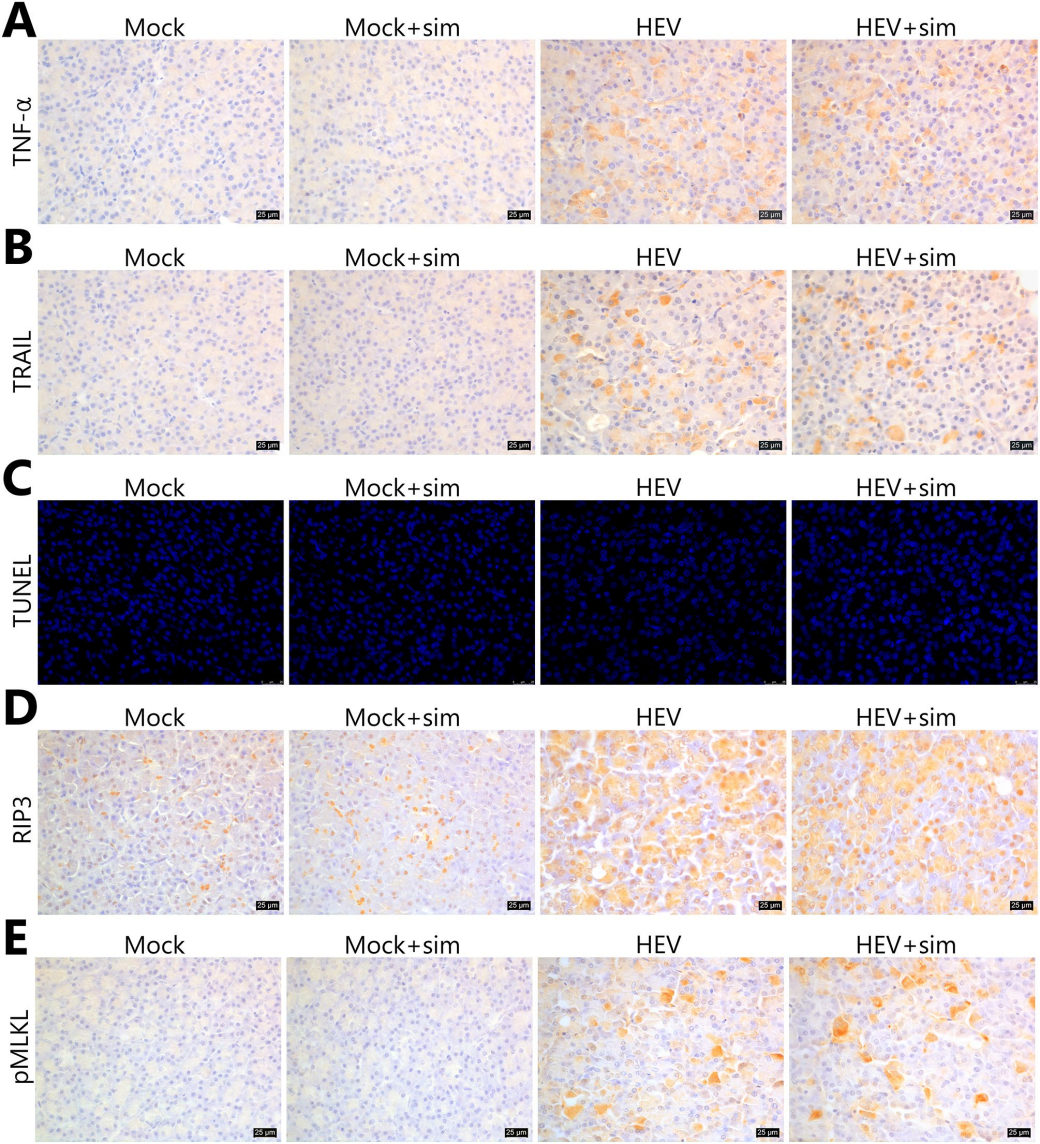
Immunohistochemical analysis of necroptosis in the pancreas. (**A**) Representative images of TNF-α staining. (**B**) Representative images of TRAIL staining. (**C**) Representative images of TUNEL assay. Green fluorescence indicates DNA fragmentation. Nuclei were counterstained with DAPI. (**D**) Representative images of RIP3 staining. (**E**) Representative images of pMLKL staining. (**A**, **B**, **D**, **E**) Nuclei were counterstained with Meyer’s haematoxylin. Scale bars = 25 μm. *HEV* hepatitis E virus, *sim* simvastatin, *TRAIL* TNF-related apoptosis-inducing ligand, *TUNEL* terminal deoxynucleotidyl transferase dUTP nick-end labelling.

Lesions in the liver and pancreas were examined for expression levels of TNF-α and TNF-related apoptosis-inducing ligand (TRAIL), which are known as ‘death ligands’. Expression patterns of the death ligands were similar to the distribution of HEV in both organs. In the liver, TNF-α expression was stronger in infiltrated lymphocytes than in hepatocytes, and TRAIL expression showed a similar pattern, but was lower than TNF-α expression (Fig. 6A,B). High levels of TNF-α and TRAIL were observed exclusively in acinar cells throughout the pancreas (Fig. 7A,B). However, although ruptured cell membranes, nuclear condensation, and high expression levels of death ligands were observed in the pancreas, terminal deoxynucleotidyl transferase dUTP nick-end labelling (TUNEL)-positive cells were not observed (Fig. 7C). In the liver, only a very weak signal, which was very low when compared to the severity of lymphocytic infiltration, was observed (Fig. 6C).

The expression of receptor-interacting protein kinase 3 (RIP3), a critical regulator of necroptosis, was investigated. A significant level of RIP3 expression was observed in hepatocytes around lymphocyte-infiltrated lesions, where RIP3 was expressed predominantly in the cytoplasm and to a lower extent in the nuclei (Fig. 6D). In the pancreas, RIP3 was strongly expressed in the nuclei and cytoplasm (Fig. 7D). The two mock groups showed mild RIP3 expression in the nuclei in the liver and pancreas. Subsequently, we examined the expression of phosphorylated mixed lineage kinase domain-like protein (pMLKL), which functions downstream of RIP3 as a key executioner of necroptosis. In the pancreas, pMLKL was predominantly expressed in morphologically changed acinar cells (Fig. 7E). RIP3-positive hepatocytes did not co-express pMLKL, and pMLKL expression was observed only in individual immune cells in the liver (Fig. 6E).

## Discussion

Although HEV infection has been well researched, much is unclear regarding its pathogenesis, for example, the transmission of HEV to the liver via the faecal-oral route^23^. Various cases with extra-hepatic symptoms of the virus have been reported, perhaps indicating an extra-hepatic replication site, but few have been experimentally proven^24–27^. Yucatan miniature pigs, which are similar to humans in anatomy and physiology, are widely used as experimental animals^28^. The structure and function of the swine immune system are more than 80% similar to those of the human immune system. Thus, the swine model may represent pathogenic and immune responses that are similar to those seen in human infectious diseases^29^. Furthermore, pigs are natural hosts of HEV gt3, and miniature pigs have been successfully infected with HEV under experimental conditions^13^. Therefore, we surmised that a miniature pig HEV infection model would be most suitable for the investigation of extra-hepatic manifestation aimed at enhancing the understanding of HEV pathogenesis.

Acute pancreatitis is a well-known complication of viral hepatitis, but the mechanism whereby it causes cell injury remains unclear^14^. HEV-induced necrotising pancreatitis has recently been reported, excluding other causes of acute pancreatitis, such as gallstones, alcohol, drugs, trauma, hypercalcemia, and hypertriglyceridemia^15^. Unlike the case for other hepatitis viruses, such as HAV, HBV, and HCV, the presence of HEV in the pancreas is unknown^15^. To date, HEV inducing necrotising pancreatitis has been genotyped in only one patient, and was identified as HEV gt1^30^. It is not known whether HEV gt1 has high tropism for the pancreas or whether it causes pancreatitis due to genetic susceptibility of the patient. The pancreas, which we focused on in our study, exhibited extensive histological changes, an abundance of HEV, and the development of necroptosis. Therefore, it may be said that this study elucidated the mechanism underlying the pancreatic injury associated with HEV infection and identified that such pancreatitis can be caused by HEV gt3 and is not limited to strains of HEV gt1. In previous studies, RIP3 expression did not match TUNEL staining, as it did in this study^31^. However, staining with propidium iodide, an indicator of cell viability based on membrane permeability, indicated strong co-localisation. Necroptosis is also termed programmed cell necrosis because it has characteristics similar to those of necrosis^31^. Thus, the indicator of cell death is more likely to be related to necrosis than to apoptosis^32^. Necroptosis can be triggered by various stimuli, the most-studied stimulus being TNF signalling^33^. When TNF-α binds to TNF receptor 1 (TNFR1), TNFR1-associated death domain protein binds to the intracellular domain of TNFR1. Subsequently, Fas-associated protein with death domain and caspase 8 are recruited to form the death-inducing signalling complex, which causes apoptosis^34^. Inhibition of caspase activity during the course of this process alternatively activates RIP3, following which RIP3 signalling phosphorylates and oligomerises MLKL downstream, resulting in necroptosis^32–34^. Further research is required to reveal the mechanism underlying caspase inhibition by HEV. Necroptosis is a recently discovered cell death pathway that is assuming importance as a topic in the field of inflammatory diseases, including acute pancreatitis^35^. Recent studies have identified necrotising pancreatitis as a major determinant of mortality associated with HEV infection^15^. This study suggests a link between necroptosis and necrotising pancreatitis caused by HEV. The presence of HEV in the pancreas sharply decreased after 21 dpi, which was in line with the pattern of faecal shedding. This suggests that pancreatic infection may be responsible for faecal shedding of HEV.

HEV is a hepatotropic virus and thus, the primary site of HEV replication is the liver. However, the path by which HEV reaches the liver remains unclear. Because inducing oral HEV infection in animal models poses difficulties, most studies focus on symptoms that develop after HEV reaches the liver^36^. In some previous studies, the HEV antigen was observed in infiltrating inflammatory cells and Kupffer cells, whereas in other studies, it was observed in the cytoplasm of hepatocytes^37–40^. In our study, HEV was continuously detected in lymphoid tissues and PBMCs, from the early stages of infection until the end of the experiment. It was rarely present in hepatocytes at 7 dpi, but multiplied in hepatocytes following liver infiltration by HEV-infected immune cells, presumably, T cells or NK cells. It was found that infected immune cells delivered HEV to hepatocytes via cell-to-cell transmission. In HEV-infected patients, NK/NKT cells showed a significant decrease in degranulation activity directly related to cytotoxicity^41^. Based on this finding, it was postulated that HEV is transmitted via impaired immunological synapses generated between infected T/NK/NKT cells and hepatocytes. However, further research is required to verify this. The liver showed no signs of cell death in the form of either apoptosis or necroptosis. Although the TUNEL assay detects only the late stages of apoptosis that follow extensive DNA fragmentation, and not the early stages of apoptosis, no satisfactory signals were detected in the liver throughout the experiment^42^. Compared with that in the pancreas, RIP3 expression was stronger in the cytoplasm of hepatocytes, although it was substantially lower in the nucleus. RIP3 is a nuclear-cytoplasmic shuttling protein that is phosphorylated in the nucleus, and nuclear export of phosphorylated RIP3 to the cytoplasm induces necrosome formation, leading to necroptosis^43^. Therefore, necroptosis of hepatocytes is not induced by insufficient nuclear–cytoplasmic export. There are several possible reasons why necroptosis does not occur in the liver. One possibility is associated with the strain and virulence of the virus. HEV gt3 used in this study may be a low-pathogenic strain as it was isolated from the faeces of pigs that did not exhibit significant clinical symptoms. In a previous study in which rabbits were inoculated with genotype 1 or 4, ALT levels increased only in rabbits inoculated with genotype 4^39^. Similarly, in chimpanzees inoculated with genotype 1, 3, or 4, only chimpanzees inoculated with genotype 4 displayed elevated ALT^11^. A second possibility relates to the dose of HEV used. Clinical and immunological progression of HEV in humans and animal models is known to be dose-dependent^36,44,45^. In this study, the amount of HEV in the liver was less than that in the pancreas and was insufficient to induce necroptosis, which may be associated with the dose–response pattern of HEV infection. This possible explanation requires verification in high-dose, long-term studies using various HEV strains.

Extra-hepatic localisation of HEV has been frequently observed. HEV RNA was localised in the immune cells of the lamina propria rather than in enterocytes in the colons of pigs naturally infected with HEV^46^. In rabbit and monkey models, HEV antigens were found in interstitial and infiltrated immune cells^39,47^. In particular, HEV antigens were detected in lymphoid tissues before they were detected in the liver. Thus, lymphoid tissue is presumed to be a replication site prior to the liver. Negative-strand-specific RT-PCR confirmed the replication of viral RNA in the tonsils and intestines in human HEV-infected pigs before it was detected in the liver^48^, which is in line with the findings in this study.

The presence of HEV in the brain has been observed in various animal models, and the possibility of in vivo and in vitro replication in neuronal cells has been reported^24,25,49^. Peripheral neurological disorders, Guillain-Barré syndrome, and neuralgic amyotrophy are strongly associated with HEV infection in humans, although this has not been experimentally substantiated^7,27^. Our study not only revealed the presence of HEV in peripheral nerves, but also indicated that HEV may be transmitted to the nervous system in a retrograde manner. These findings were obtained following intravenous virus inoculation, which has several advantages over the faecal–oral route. When HEV enters the host orally, it replicates in the primary site and then spreads to various target organs. This process takes a long time, during which the host cells initiate various immune responses. In contrast, upon intravenous administration, which results in a high virus titre in the blood from the early stage of infection, the virus would have spread more easily. However, our findings provide a rationale for the distribution of HEV in the body and the pathogenesis associated with cell damage caused thereby.

Simvastatin administered for immunosuppression and efficient replication of HEV significantly affected only the IFN-γ levels. IFN-γ levels increased only in the HEV + sim group at 28 dpi, whereas it was hardly expressed in the other groups. This result was presumably associated with the activation of NK/NKT cells during HEV infection. NK/NKT cells reportedly are rapidly activated in patients with acute HEV infection^50^. In addition, HCV activates NK cells by consuming IFN-γ^51^. HEV also consumes IFN-γ during the activation of NK/NKT cells. However, IFN-γ levels were elevated in the HEV + sim group, suggesting that simvastatin reduced IFN-γ-induced MHC class II expression on antigen-presenting cells, thereby lowering the NK/NKT cell activity^52–54^. Simvastatin treatment increased human norovirus infectivity in gnotobiotic pig models, possibly by inhibiting innate immunity and lowering cholesterol^55^. However, the effect of simvastatin on HEV infection in the miniature pig model was limited, presumably due to differences in the infection mechanisms of HEV and norovirus.

In conclusion, the present study showed that HEV gt3 causes pancreatitis in pigs and that injury to pancreatic cells is associated with necroptosis. In addition, HEV is distributed to immune cells in various organs, including the liver. The miniature pig HEV infection model appears to be more suitable for studying extra-hepatic manifestations than for investigating hepatitis caused by HEV.

## Materials and methods

### Virus inoculum

HEV gt3 (GenBank accession no.: MT007930) was obtained from stool samples of naturally infected domestic pigs in Chungcheongnam-do, Korea. A HEV gt3 stool suspension was filtered sequentially through 0.45-µm- and 0.20-µm-pore syringe filters. Sterility was confirmed by incubating the filtered suspension on sheep blood agar plates. The inoculum was tested for the absence of enteroviruses that may cause pancreatitis using RT-qPCR^20^, and was stored at − 70 °C following titration.

### Experimental design

Thirty 8-week-old SPF Yucatan miniature pigs were tested for the HEV antigen and the most significant pig viral pathogens (porcine reproductive and respiratory syndrome virus, classical swine fever virus, swine influenza virus, Aujeszky’s disease virus, porcine circovirus type 2) using ELISA. Pigs were divided into four groups: (i) group HEV (n = 12) was intravenously injected with 1.2 × 10^6^ genome equivalents of HEV gt3; (ii) group HEV + simvastatin (HEV + sim; n = 12) was administered 8 mg/kg of simvastatin via the oral route for 6 days prior to virus inoculation, intravenously infected with 1.2 × 10^6^ genome equivalents of HEV gt3, and administered 4 mg/kg simvastatin for 5 more days following inoculation; (iii) group mock + simvastatin (mock + sim; n = 3) was treated in a manner similar to group HEV + sim, except for HEV inoculation; and (iv) group mock infection (mock; n = 3) was treated with PBS^21^. All experimental methods were approved by the Chung-ang University Institutional Animal Care and Use Committee (approval number: 2016-00071). All experiments were performed in accordance with relevant guidelines and regulations.

Plasma and PBMCs were extracted from whole blood collected in heparin tubes using a Lymphoprep density gradient centrifuge (Nyegaard A/S, Oslo, Norway). Stool samples were diluted tenfold with PBS and total RNA was immediately extracted. Tissue samples were formalin-fixed and paraffin-embedded.

### RT-qPCR

RT-qPCR was used to detect HEV RNA in faeces, plasma, and PBMCs. The qPCR mixture (25 μl) consisted of 9 μl of nuclease-free water, 12.5 μl of Premix Ex Taq (2 ×), forward and reverse primers at 400 nM each, probes at 200 nM, and 1 μl of cDNA. RT-qPCRs were run in a Thermal Cycler Dice Real Time System (TaKaRa, Shiga, Japan)^22^.

### Determination of ALT and AST concentrations

ALT and AST concentrations in plasma were measured on the day of collection using International Federation of Clinical Chemistry (IFCC) methods, on a 7,020 automatic analyser (Hitachi, Tokyo, Japan).

### ELISAs for evaluating IFN levels and seroconversion

IFN-α and -γ expression and seroconversion were determined by ELISA, using a Porcine Interferon α ELISA Kit (Cusabio, Balitmore, MD, USA), a Porcine Interferon gamma ELISA Kit (Thermo Fisher, Waltham, MA, USA), and an HEV antibody ELISA Kit (MP Biomedicals, Solon, OH, USA).

### Histopathology and IHC

For histopathological examination purposes, tissue slides were stained using haematoxylin and eosin. For IHC, slides were deparaffinised and rehydrated, washed, and subjected to antigen retrieval. Endogenous peroxidase was quenched and non-specific immunoreactivity was blocked using normal serum. Then, the sections were incubated with primary antibodies at room temperature for 1 h. After washing, the sections were incubated with biotinylated secondary antibodies, rinsed, and incubated with avidin–biotin-HRP complex (Vector Laboratories). To visualise immunoreaction sites, the sections were incubated with DAB solution (Vector Laboratories) and counterstained with Mayer’s haematoxylin, dehydrated, clarified, and sealed using permanent mounting medium under coverslips.

### TUNEL assay

Apoptotic cells in the liver and pancreas were detected by TUNEL assays, using a cell apoptosis detection kit (In Situ Death Detection kit, Fluorescein; Roche Applied Science, Mannheim, Germany). Nuclei of apoptotic cells emitted green fluorescence, and normal cells were counterstained with DAPI.

### Statistical analysis

All data are expressed as the means ± SDs. Statistical analysis was performed using a one-way ANOVA followed by Bonferroni’s post-hoc tests in SPSS version 23 (IBM, NY, USA). A p-value < 0.05 was considered statistically significant.

## Supplementary information

Supplementary information
