# Supplementary material for: Experimental infection of hepatitis E virus induces pancreatic necroptosis in miniature pigs
Source: Sci Rep. 2020 Jul 21;10:12022. doi: 10.1038/s41598-020-68959-3 (PMC7374588; doi:10.1038/s41598-020-68959-3)
Supplement: Supplementary file 1 — Supplementary information [file 41598_2020_68959_MOESM1_ESM.docx]

**Experimental infection of hepatitis E virus induces pancreatic necroptosis in miniature pigs**

Soontag Jung, Dong Joo Seo, Daseul Yeo, Zhaoqi Wang, Ae Min, Ziwei Zhao, Mengxiao Song, In-Soo Choi, Jinjong Myoung, Changsun Choi

*Corresponding author: Changsun Choi, D.V.M., Ph.D.

Department of Food and Nutrition, School of Food Science and Technology

College of Biotechnology and Natural Resources

Chung-Ang University, Anseong-Si, Gyeonggi-do 17546, Republic of Korea

Telephone: +82-31-670-4589, Fax: +82-31-676-8741, E-mail address: [cchoi@cau.ac.kr](about:blank)

Table of contents

[Table S1. 6](#_Toc43049025)

[Figure S1. 7](#_Toc43049026)

[Figure S2. 8](#_Toc43049027)

[Figure S3. 9](#_Toc43049028)

[Figure S4. 10](#_Toc43049029)

[Table S2. 11](#_Toc43049030)

**Materials and Methods**

**Experimental design**

Thirty 8-week-old SPF Yucatan miniature pigs were tested for the HEV antigen and the most significant pig viral pathogens (porcine reproductive and respiratory syndrome virus, classical swine fever virus, swine influenza virus, Aujeszky’s disease virus, porcine circovirus type 2) using ELISA. Pigs were divided into four groups: (i) group HEV (n = 12) was intravenously injected with 1.2 × 10^6^ genome equivalents of HEV gt3; (ii) group HEV+simvastatin (HEV+sim; n = 12) was administered 8 mg/kg of simvastatin via the oral route for 6 days prior to virus inoculation, intravenously infected with 1.2 × 10^6^ genome equivalents of HEV gt3, and administered 4 mg/kg simvastatin for 5 more days following inoculation; (iii) group mock+simvastatin (mock+sim; n = 3) was treated in a manner similar to that for group HEV+sim, except for HEV inoculation; and (iv) group mock infection (mock; n = 3) was treated with PBS. Blood and rectal swab samples were collected at 0, 3, 7, 10, 14, 17, 21, 24, and 28 days post inoculation (dpi). HEV-inoculated groups were necropsied weekly to collect liver, pancreas, brain, heart, lung, kidney, tonsil, thymus, spleen, lymph node, stomach, duodenum, jejunum, ileum, and colon tissues for histopathological examination, whereas the control groups were necropsied on day 28. All experimental methods were approved by the Chung-ang University Institutional Animal Care and Use Committee (Approval number: 2016-00071).

Plasma and peripheral blood mononuclear cells (PBMCs) were extracted from whole blood collected in heparin tubes using a Lymphoprep density gradient centrifuge (Nyegaard A/S, Oslo, Norway). Stool samples were suspended in a solution diluted 10-fold with PBS, and total RNA was immediately extracted. Fresh tissue samples were fixed immediately in 10% neutral buffered formalin solution. Fixed tissues were dehydrated, clarified, and embedded in paraffin blocks. Four-micrometre-thick sections were cut and mounted on silane-coated slides.

**Quantitative reverse transcription PCR (RT-qPCR)**

Total RNA was extracted from faeces and PBMCs using an RNeasy Mini kit (Qiagen, Hilden, Germany) and from plasma using a NucleoSpin® Virus kit (Macherey & Nagel, Dueren, Germany). The RT mixture (10 μl) included 1 μl of 10× PCR buffer, 1 μl of 10 mM dNTP, 0.5 μl of 50 μM oligo d(T)_16_, 10 units RNase inhibitor, 25 units MuLV reverse transcriptase, and 1 μl of total RNA. RT was carried out at 42°C for 15 min, and enzymes were inactivated at 95°C for 5 min.

RT-qPCR was used to detect HEV RNA in the faeces, plasma, and PBMCs. qPCRs were run in 25-μl reaction mixtures comprising 9 μl of nuclease-free water, 12.5 μl of Premix Ex Taq (2×), forward (JVHEVF, 5′-GGT GGT TTC TGG GGT GAC-3′) and reverse primers (JVHEVR, 5′-AGG GGT TGG TTG GAT GAA-3′) at 400 nM each, probe (JVHEVP, 5′-FAM-TGA TTC TCA GCC CTT CGC-TAMRA-3′) at 200 nM, and 1 μl of cDNA, in a Thermal Cycler Dice Real Time System (TaKaRa, Shiga, Japan). The thermal cycles were as follows: initial denaturation at 95°C for 5 min, 45 cycles at 95°C for 10 s, 55°C for 20 s, and 72°C for 15 s.

**ELISA for evaluating interferon (IFN) levels and seroconversion**

IFN-α and -γ expression and seroconversion were analysed by ELISA, using a Porcine Interferon α ELISA Kit (CSB-E07328p; Cusabio, Balitmore, MD, USA), a Porcine Interferon gamma ELISA Kit (EPINFG1; Thermo Fisher, Waltham, MA, USA), and an HEV antibody ELISA Kit (0763541096; MP Biomedicals, Solon, OH, USA). According to the manufacturers’ standard protocols, IFN-α, IFN-γ, and seroconversion were detected in plasma samples from the four groups described in the experimental design. Absorbance was read at 450 nm on an Epoch spectrophotometer (BioTek, Winooski, VT, USA). Quantitative analysis was performed using a standard curve. The cut-off value was calculated as 0.5 plus mean absorbance of the non-reactive control, according to the manufacturers’ instructions. A plasma sample showing an absorbance value greater than the cut-off value was considered to be positive.

**Histopathology and immunohistochemistry (IHC)**

For histopathological examination purposes, tissue slides were stained using haematoxylin and eosin according to standard protocols. For IHC, slides were deparaffinised and rehydrated, washed in TBS-T, and subjected to antigen retrieval. After endogenous peroxidase was quenched using BLOXALL solution (SP-6000; Vector Laboratories, Burlingame, CA, USA) for 10 min, non-specific immunoreactivity was blocked with 1.5% normal serum for 30 min. Then, the sections were incubated with primary antibodies for HEV (70R-HR003; Fitzgerald, 1:700), CD3ε (sc-1127; Santa Cruz, 1:100), CD19 (orb251475; Biorbyt, 1:100), CD107a (MCA2315GA; Bio-Rad, 1:100), CD163 (MCA2311GA; Bio-Rad, 1:100), TRAIL (sc-6079; Santa Cruz, 1:100), TNF-α (MAB6903; R&D systems, 1:100) at room temperature for 1 h. After washing with TBS-T, the sections were incubated with biotinylated secondary antibodies (Vector Laboratories) for 30 min, rinsed, and incubated with avidin-biotin-HRP complex (Vector Laboratories). To visualise immunoreaction sites, the sections were incubated with DAB solution (Vector Laboratories) and counterstained with Mayer’s haematoxylin, dehydrated, clarified, and sealed using permanent mounting medium under coverslips.

Table S1. Faecal shedding and cell-free and cell-associated viremia in individual pigs infected with HEV gt3.

HEV, Hepatitis E virus; sim, simvastatin; n/a, not applicable

| Group | No. | Positive (+) or negative (–) for HEV RNA detection in the plasma/PBMC/stool on the indicated day post inoculation | | | | | | | | |
| --- | --- | --- | --- | --- | --- | --- | --- | --- | --- | --- |
|  |  | 0 | 3 | 7 | 10 | 14 | 17 | 21 | 24 | 28 |
| HEV | 1 | –/–/– | –/–/– | –/–/+ | n/a | n/a | n/a | n/a | n/a | n/a |
|  | 2 | –/–/– | –/+/+ | –/+/+ | n/a | n/a | n/a | n/a | n/a | n/a |
|  | 3 | –/–/– | –/–/– | –/+/– | n/a | n/a | n/a | n/a | n/a | n/a |
|  | 4 | –/–/– | –/–/– | –/–/+ | –/–/+ | –/–/+ | n/a | n/a | n/a | n/a |
|  | 5 | –/–/– | –/–/– | –/–/+ | –/+/– | –/+/+ | n/a | n/a | n/a | n/a |
|  | 6 | –/–/– | –/–/+ | –/–/+ | –/–/+ | –/+/+ | n/a | n/a | n/a | n/a |
|  | 7 | –/–/– | –/–/– | –/–/+ | –/+/+ | –/+/+ | –/–/– | –/+/+ | n/a | n/a |
|  | 8 | –/–/– | –/+/– | –/+/+ | –/+/+ | –/–/+ | –/+/+ | –/–/– | n/a | n/a |
|  | 9 | –/–/– | –/+/+ | –/+/+ | –/+/– | –/+/+ | –/–/+ | –/–/+ | n/a | n/a |
|  | 10 | –/–/– | –/+/– | –/+/+ | –/+/+ | –/+/+ | –/+/+ | –/–/– | –/+/– | –/+/– |
|  | 11 | –/–/– | –/+/– | –/+/+ | –/+/+ | –/+/+ | –/–/+ | –/+/+ | –/–/+ | –/+/– |
|  | 12 | –/–/– | –/+/+ | –/–/+ | –/+/+ | –/–/– | –/+/– | –/+/– | –/+/– | –/+/– |
| HEV  +sim | 13 | –/–/– | –/–/+ | –/+/+ | n/a | n/a | n/a | n/a | n/a | n/a |
|  | 14 | –/–/– | –/+/– | –/–/+ | n/a | n/a | n/a | n/a | n/a | n/a |
|  | 15 | –/–/– | –/–/– | –/–/+ | n/a | n/a | n/a | n/a | n/a | n/a |
|  | 16 | –/–/– | –/–/– | –/–/+ | –/+/– | –/–/+ | n/a | n/a | n/a | n/a |
|  | 17 | –/–/– | –/–/+ | –/–/+ | –/+/– | –/+/+ | n/a | n/a | n/a | n/a |
|  | 18 | –/–/– | –/+/+ | –/+/– | –/+/– | –/+/+ | n/a | n/a | n/a | n/a |
|  | 19 | –/–/– | –/+/+ | –/+/+ | –/+/+ | –/+/+ | –/+/+ | –/+/+ | n/a | n/a |
|  | 20 | –/–/– | –/+/+ | –/+/+ | –/+/+ | –/+/+ | –/–/+ | –/+/+ | n/a | n/a |
|  | 21 | –/–/– | –/–/– | –/+/– | –/–/+ | –/+/– | –/+/– | –/–/+ | n/a | n/a |
|  | 22 | –/–/– | –/+/– | –/+/+ | –/+/+ | –/+/+ | –/+/+ | –/–/– | –/–/– | –/+/– |
|  | 23 | –/–/– | –/+/– | –/+/– | –/+/+ | –/+/+ | –/+/+ | –/+/+ | –/–/– | –/+/– |
|  | 24 | –/–/– | –/+/– | –/+/+ | –/–/+ | –/+/+ | –/–/+ | –/+/– | –/+/+ | –/+/– |
| Mock | 25 | –/–/– | –/–/– | –/–/– | –/–/– | –/–/– | –/–/– | –/–/– | –/–/– | –/–/– |
|  | 26 | –/–/– | –/–/– | –/–/– | –/–/– | –/–/– | –/–/– | –/–/– | –/–/– | –/–/– |
|  | 27 | –/–/– | –/–/– | –/–/– | –/–/– | –/–/– | –/–/– | –/–/– | –/–/– | –/–/– |
| Mock+sim | 28 | –/–/– | –/–/– | –/–/– | –/–/– | –/–/– | –/–/– | –/–/– | –/–/– | –/–/– |
|  | 29 | –/–/– | –/–/– | –/–/– | –/–/– | –/–/– | –/–/– | –/–/– | –/–/– | –/–/– |
|  | 30 | –/–/– | –/–/– | –/–/– | –/–/– | –/–/– | –/–/– | –/–/– | –/–/– | –/–/– |


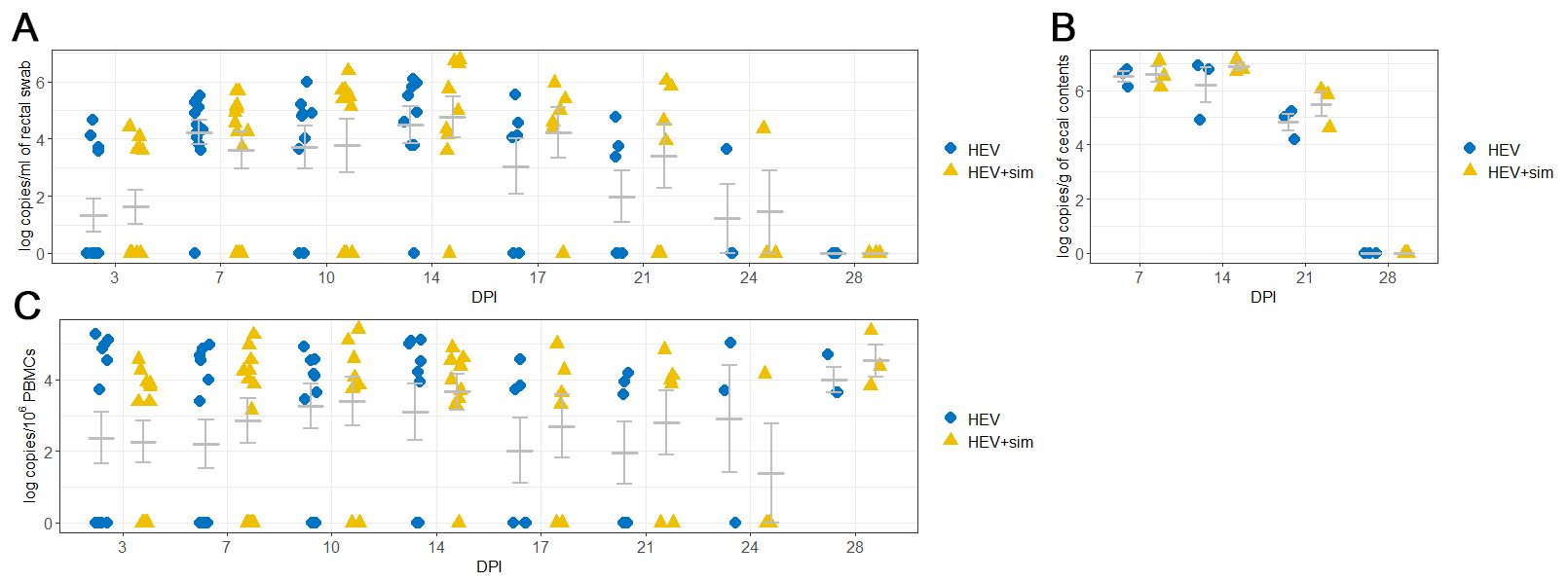


Figure S1. Viral RNA titres in samples from pigs infected with HEV. Time course of viral RNA titres in rectal swabs (A), caecal contents (B), and PBMCs (C) in pigs infected with HEV gt3. HEV, hepatitis E virus; sim, simvastatin.


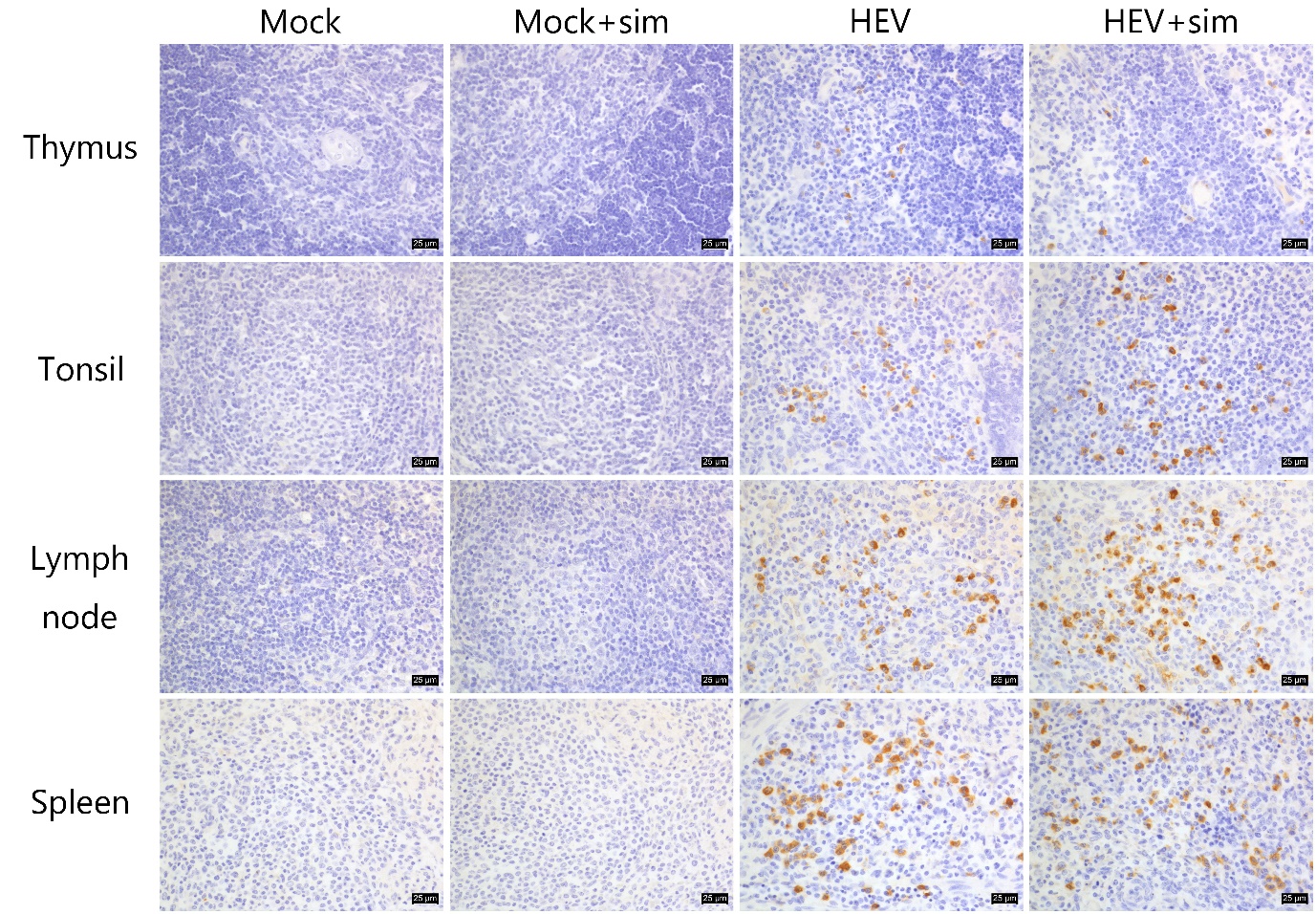


Figure S2. Localisation of HEV in lymphoid tissues. Brown colour indicates HEV. Nuclei were visualised by counterstaining with Meyer’s haematoxylin. Scale bars = 25 μm. HEV, hepatitis E virus; sim, simvastatin.


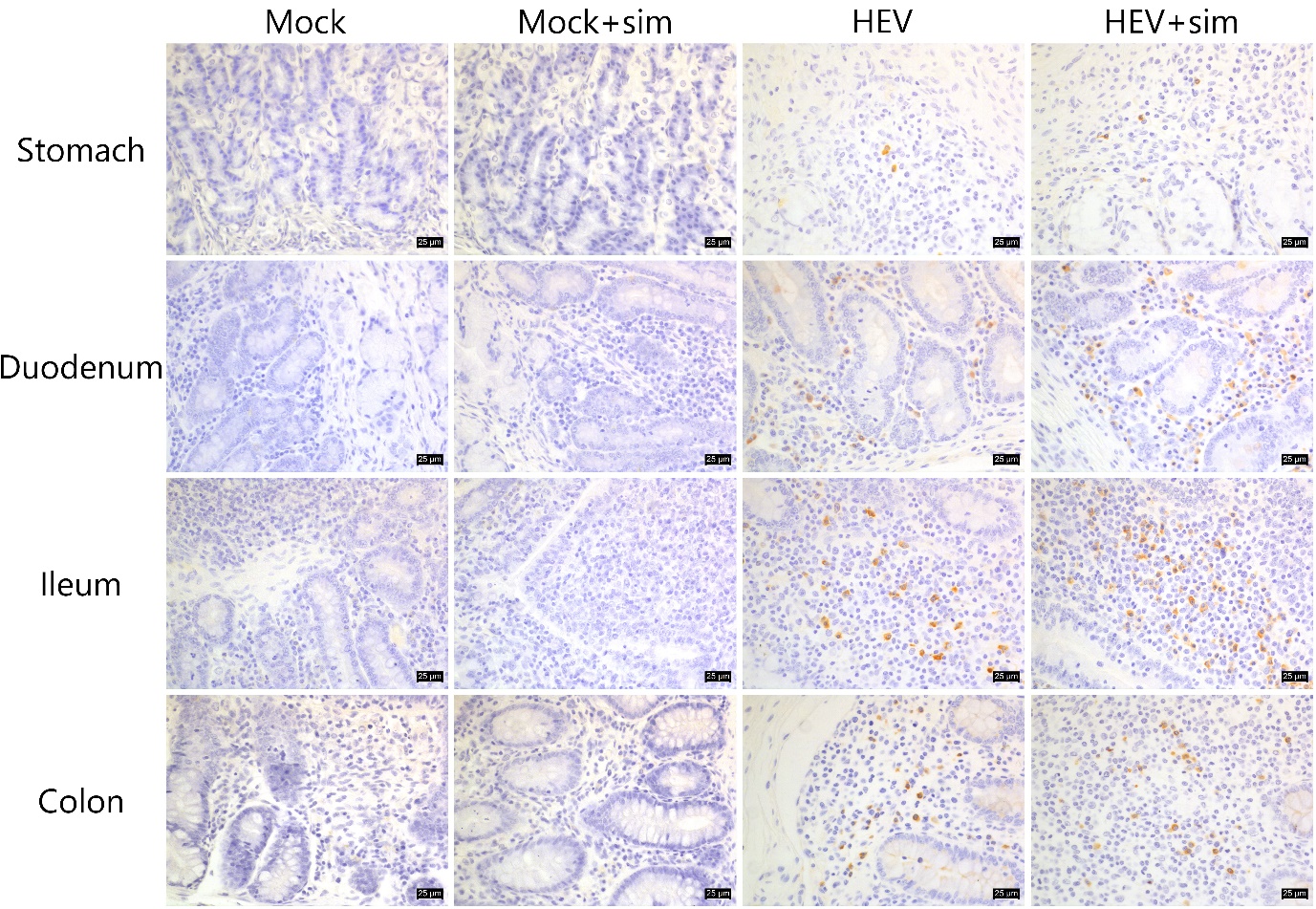


Figure S3. Localisation of HEV in the gastrointestinal tract. Brown colour indicates HEV. Nuclei were visualised by counterstaining with Meyer’s haematoxylin. Scale bars = 25 μm. HEV, hepatitis E virus; sim, simvastatin.


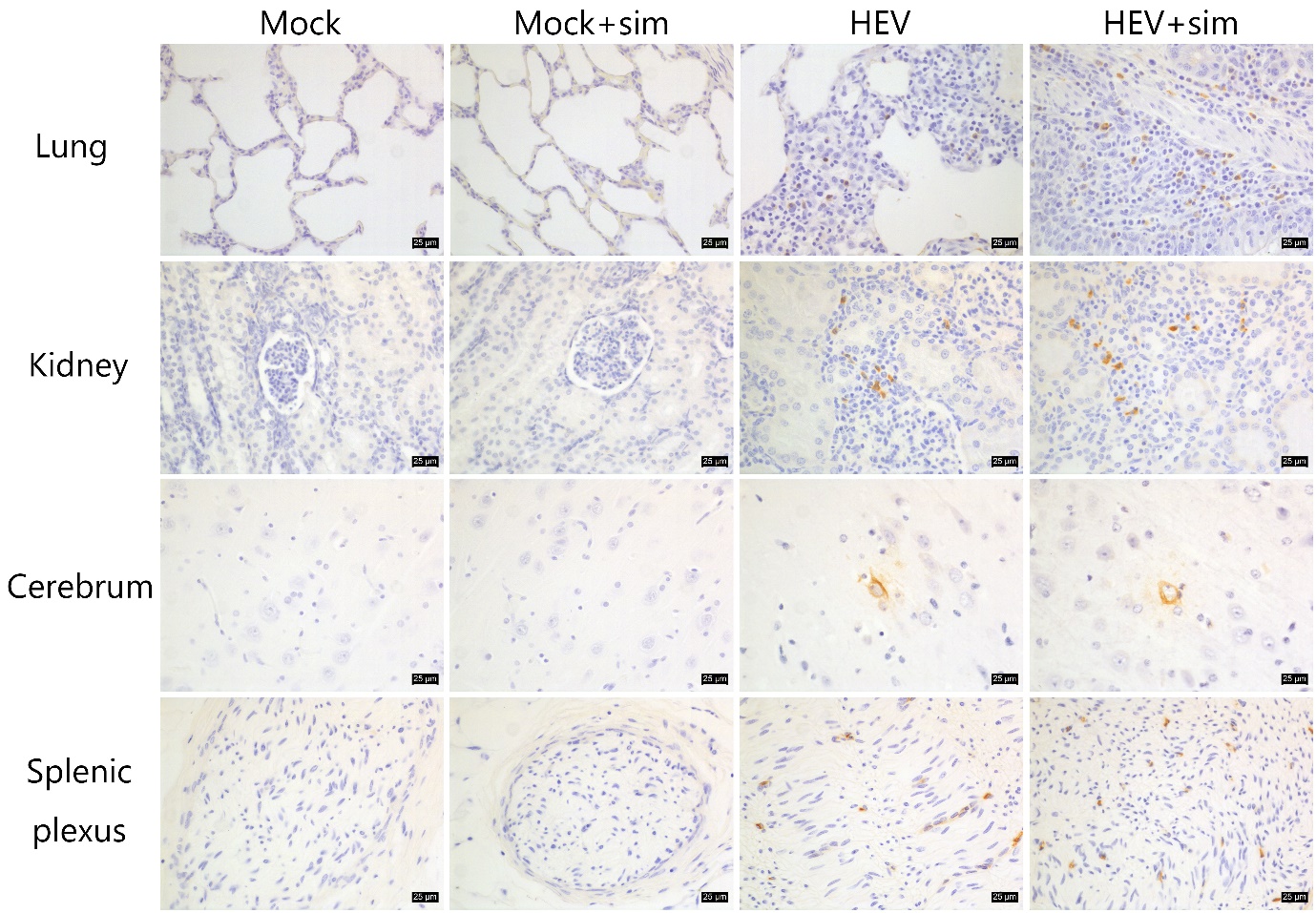


Figure S4. Localisation of HEV in the lungs, kidneys, and nerve tissues. The brown colour indicates HEV. Nuclei were visualised by counterstaining with Meyer’s haematoxylin. Scale bars = 25 μm. HEV, hepatitis E virus; sim, simvastatin.

Table S2. Immunohistochemistry grades of HEV-infected cells in various tissues.

| Tissue | | Group | IHC grades^a^ for individual pigs (n = 3) | | | |
| --- | --- | --- | --- | --- | --- | --- |
|  | |  | 7 dpi | 14 dpi | 21 dpi | 28 dpi |
| Liver | Hepatocytes | HEV | +/+/+ | +/+/+ | +/+/+ | ++/++/+ |
|  |  | HEV+sim | +/+/+ | +/+/+ | +/++/++ | ++/+++/+++ |
|  | Infiltrated cells | HEV | +/+/+ | +/+/+ | ++/+/++ | +/+/+ |
|  |  | HEV+sim | +/+/+ | +/++/+ | +++/+++/++ | +/+/++ |
| Pancreas | | HEV | ++/+/++ | ++/+/+ | ++/++/+ | +/+/+ |
|  |  | HEV+sim | +/+/+++ | +/++/++ | +++/++/+++ | +/+/+++ |
| Kidneys | | HEV | +/+/+ | +/+/+ | +/+/+ | +/+/+ |
|  |  | HEV+sim | +/+/+ | +/+/+ | +/+/+ | +/+/+ |
| Splenic plexus | | HEV | +/+/+ | +/+/+ | +/+/+ | –/–/– |
|  |  | HEV+sim | +/+/+ | +/+/+ | +/+/+ | –/–/– |
| Cerebrum | | HEV | –/–/– | –/–/+ | +/+/+ | +/–/+ |
|  |  | HEV+sim | –/+/– | +/–/– | -/+/+ | +/–/– |
| Cerebellum | | HEV | –/–/– | –/+/– | –/–/– | –/–/– |
|  |  | HEV+sim | –/–/– | –/–/– | –/+/+ | +/–/– |
| Thymus | | HEV | –/+/– | –/+/+ | +/+/– | –/+/+ |
|  |  | HEV+sim | –/–/– | +/–/– | –/+/+ | –/+/+ |
| Tonsil | | HEV | +/+/+ | +/+/++ | ++/++/+ | +/++/++ |
|  |  | HEV+sim | +/++/++ | ++/+/++ | ++/++/++ | +/++/++ |
| Lymph nodes | | HEV | ++/++/+ | ++/++/+++ | ++/+++/++ | ++/+++/++ |
|  |  | HEV+sim | ++/+++/+++ | ++/+++/+++ | +++/+++/+++ | +++/+++/+++ |
| Spleen | | HEV | ++/++/++ | ++/+++/++ | ++/+/++ | +/+/++ |
|  |  | HEV+sim | +++/+++/++ | ++/++/++ | ++/++/++ | ++/++/++ |
| Stomach | | HEV | –/+/+ | –/+/+ | +/+/+ | –/+/+ |
|  |  | HEV+sim | +/–/– | –/+/+ | +/+/+ | +/+/– |
| Duodenum | | HEV | +/+/++ | +/+/++ | ++/++/++ | ++/+++/+++ |
|  |  | HEV+sim | ++/+/+ | ++/++/++ | +++/++/+++ | +++/++/+++ |
| Jejunum | | HEV | –/–/– | –/–/– | –/–/– | –/–/– |
|  |  | HEV+sim | –/–/– | –/–/– | –/–/– | –/–/– |
| Ileum | | HEV | +/+/+ | ++/++/+ | ++/+/+ | +/++/+ |
|  |  | HEV+sim | +/+/+ | +/++/+ | +/++/+ | ++/++/++ |
| Colon | | HEV | +/+/+ | +/+/+ | +/+/+ | +/+/+ |
|  |  | HEV+sim | ++/+/+ | +/+/+ | +/++/+ | +/++/+ |
| Lungs | | HEV | +/+/– | +/+/– | –/–/– | +/+/– |
|  |  | HEV+sim | +/+/+ | +/+/– | –/+/+ | +/++/– |
| Heart | | HEV | –/–/– | –/–/– | –/–/– | –/–/– |
|  |  | HEV+sim | –/–/– | –/–/– | –/–/– | –/–/– |

^a^ HEV-infected cell proportions were scored as follows: –, none; +, <5 cells/high-power field (400×); ++, 5–10 cells/high-power field; +++, >10 cells/high-power field. HEV, hepatitis E virus; dpi, day post inoculation; sim, simvastatin.
